# Phase Unwrapping and Frequency Points Subdivision of the Frequency Sweeping Interferometry Based Absolute Ranging System

**DOI:** 10.3390/s22082904

**Published:** 2022-04-10

**Authors:** Luming Song, Guang Shi, Hong Liu, Hongyi Lin, Fumin Zhang, Dong Sun

**Affiliations:** 1 School of Optoelectronic and Communication Engineering, Xiamen University of Technology, Xiamen 361024, China; jerrysong97@hotmail.com (L.S.); eedlh@163.com (H.L.); linyi0714@163.com (H.L.); 2 School of Mechanical Engineering, Hangzhou Dianzi University, Hangzhou 310018, China; shiguang@hdu.edu.cn; 3 State Key Laboratory of Precision Measurement Technology and Instruments, Tianjin University, Tianjin 300072, China; zhangfumin@tju.edu.cn

**Keywords:** absolute distance measurement, FSI, HT, CZT

## Abstract

Frequency sweeping interferometry (FSI) based absolute distance ranging has high precision and no ranging blind area. It can be used to realize large-scale and non-cooperative target measurement. However, the nonlinear frequency modulation of the laser seriously affects the ranging accuracy. In this manuscript, a measurement method assisted by Hilbert Transform (HT) and Chirp-z Transform (CZT) is proposed, which can realize the phase unwrapping of the beat signal, the length reduction in the delay fiber of auxiliary optical path, and the improvement of the frequency resolution. The narrow-band frequency suitable for HT is further studied. In the experiment, the ranging resolution is 70 μm and the standard deviation is 12.6 μm within a distance of 4005 mm.

## 1. Introduction

With the dominance of no ranging blind area, non-cooperative target, and high signal to noise ratio, the FSI is coming into prominence in the field of engineering application, such as optical coherence tomography [1], the measurement of three-dimensional coordinate [2] and parameters of rotors [3], important equipment manufacturing [4] and satellite formation [5]. FSI is based on the Michelson interferometer, and the distance is calculated by the beat frequency caused by the sweeping frequency and phase difference [6,7]. Hence, the precision of FSI in absolute distance measurement will rely much on the tunability of its laser source [8]. However, the hysteric response of piezoelectric (PZT) causes the frequency nonlinear tuning of the external cavity diode laser (ECDL), which makes the phase error of the interferometry signal [9,10,11,12].

Nonlinear frequency sweeping is suppressed by upgrading hardware or using a software algorithm [13,14,15]. Photoelectric crystal [16], phase-locked loop (PLL) [17], and other technologies are used for active frequency stabilization. Iiyama et al. [17] obtained the frequency difference between the reference signal and the heterodyne interference signal by a lock-in amplifier. After PLL modulation, the improvement of the linearity was estimated to be about 10 dB. Greiner et al. [16] added an intracavity electro-optic crystal into the resonator for frequency stabilization. The current-induced frequency modulation noise was reduced two orders of magnitude. Kakuma [18] marked the sweeping frequency of the laser with the special absorption frequency of the Rb cell. The gradient of interference fringes was accurately determined by the linear least square fitting method. Theoretically, the ranging accuracy was greater than one quarter of the wavelength. These methods are based on upgrading of hardware, which makes the ranging system more complex.

Furthermore, several algorithms are used to correct the nonlinear frequency sweeping [19,20]. Shi et al. [21] resampled the beat signal in the measurement optical path with the peak-valley resampling method, which realized the linearization of the frequency ramp. The distance spectrum of the resampled signal was obtained by the fast Fourier transform (FFT), and the measurement precision was enhanced to 50 μm within 8.7 m. Liu et al. [22] tracked the frequency of interference signal with an extended Kalman filter. The relative phase extraction error in the fractional part is <1.5% and the standard deviation of absolute distance measurement is <2.4 μm. In order to satisfy the equal frequency interval sampling, sampled points are often selected at the peak and valley positions of the auxiliary path interference signal. To satisfy the Nyquist sampling theorem, the optical path difference of the auxiliary optical path needs to be twice as long as that of the measurement optical path. This puts forward a strict demand on the length of the delay fiber of the auxiliary optical path. In addition, the all-phase FFT is used to calculate the frequency information of the beat signal. Since the accuracy of FFT is related to the number of points of the signal, the more points involved in FFT, the higher accuracy of FFT, which is usually set to $2^{N}$ points. This reduces the calculation speed and wastes a large number of information points.

Moreover, an optical frequency comb can calibrate the sweeping frequency of a continuous wave (CW) laser [23]. Jia et al. [24] corrected the nonlinearity by sampling the ranging signals at equal frequency intervals with a microresonator soliton comb. However, this measurement method is not suitable for industrial environments because of its complex structure and considerately high cost [25].

In this manuscript, we expand the phase of the beat signal of the auxiliary optical path by HT. The number of sampled points is increased with equal frequency interval in a period, and the peak frequency of the resampled signal is refined by CZT. Compared with FFT, the advantage of CZT is that only M points near the peak frequency need to be calculated, which greatly reduces the calculated points and improves the calculation speed.

## 2. Methodology

**Figure 1:**
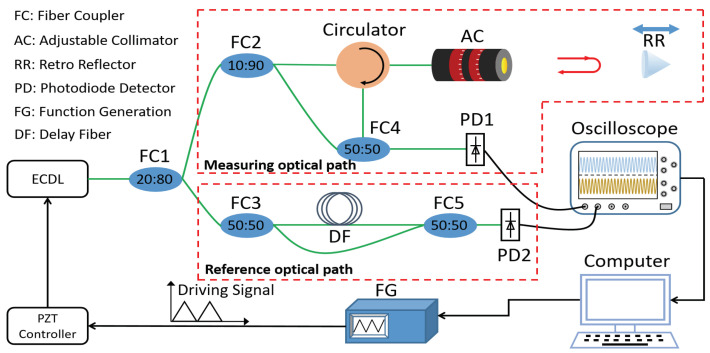
Schematic diagram of FSI ranging system.

Figure 1 shows a schematic diagram of the FSI ranging system. The triangular wave signal produced by the signal generator is transmitted to the PZT, which is used to modulate the frequency. The laser emitted by ECDL is separated by the fiber coupler (FC1). Furthermore, 80% of the optical power passes through the measurement optical path and 20% of the optical power passes through the auxiliary optical path. The optical power of the measurement optical path is divided into two beams by the fiber coupler (FC2); 90% of the optical power is incident to the retro reflector (RR) and the reflected light is combined with the local oscillator optical power (10%) at the 50:50 fiber coupler (FC4).

The sweeping frequency (*f*), the light intensity of the beat signal ($I_{mea}$), and the relationship between the time delay ($\tau_{0}$) and measurement distance (L) can be expressed as Equations (1)–(3) [26],

(1) $$\begin{array}{c} f=at+f_{0} \end{array}$$

(2) $$\begin{array}{c} I_{mea}=I_{0}\cos\left[2\pi \left(f_{0}\tau_{0}+at\tau_{0}\right)\right] \end{array}$$

(3) $$\begin{array}{c} L=\frac{\tau_{0}c}{2n} \end{array}$$

where, *a* is the frequency variation of laser, $f_{0}$ is the initial frequency, *n* is the refraction index, *c* is the speed of light, *t* is the time, and $I_{0}$ is the normalized light intensity.

Since the modulated signal is a triangular wave, the frequency of the beat signal is a fixed value equals to 2$a\tau_{0}$. Thus, Equation (3) can be rewritten as,

(4) $$\begin{array}{c} L=\frac{f_{beat}c}{4an} \end{array}$$

where $f_{beat}$ is the frequency of the beat signal. Similarly, in the auxiliary optical path, the optical power is divided into two parts by the 50:50 fiber coupler (FC3) which are merged at the 50:50 fiber coupler (FC5). The time delay ($\tau_{r}$) in the auxiliary optical path is related to the length of the delay fiber; the beat signal of the auxiliary optical path ($I_{ref}$) can be expressed as Equation (5).

(5) $$\begin{array}{c} I_{ref}=I_{0}\cos\left[2\pi \left(f_{0}\tau_{r}+at\tau_{r}\right)\right] \end{array}$$

The higher-order expansion of the actual frequency variation of the laser can be expressed as $a^{\prime }=a_{0}+\sum_{i=1}^{R}a_{i}t^{i}\tau_{0}$, where $a_{i}$ is the nonlinear frequency modulation coefficient, R is the high-order term of nonlinear frequency modulation, and $a_{0}$ is the slope of frequency modulation. Equation (2) can be expressed as,

(6) $$\begin{array}{c} I_{mea}=I_{0}\cos\left[2\pi \left(f_{0}\tau_{0}+a_{0}t\tau_{0}+\sum_{i=1}^{R}a_{i}t^{i}\tau_{0}\right)\right] \end{array}$$

**Figure 2:**
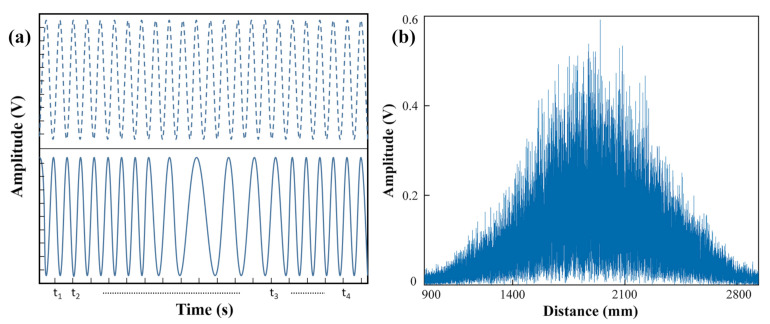
Nonlinear frequency modulation. (**a**) the beat signal comparison, (**b**) widened spectrum (FWHM) of beat signal.

As shown in Figure 2a, the blue dot curve represents an ideal beat signal with a fixed period, and the blue solid curve is the actual beat signal with the unstable period. Figure 2b shows the distance spectrum without resampling. The full width at half maxima (FWHM) represents the ranging resolution, which is seriously widened. Thus, the effective distance cannot be obtained from Figure 2b.

In order to suppress the nonlinear frequency modulation of the laser, we built an auxiliary optical path in the FSI system, which is an auxiliary interferometer with a fixed delay fiber. The beat signal of the auxiliary optical path is used to resample the beat signal of the measurement optical path, and the resampled signal ($I_{sam}$) is given by Equation (7),

(7) $$\begin{array}{c} I_{sam}=I_{0}\cos\left(2\pi \frac{N}{2\tau_{r}}\tau_{0}\right) \end{array}$$

where *N* is the resample point at the equal frequency interval of π; the frequency of resample ($F_{s}^{\prime }$) can be expressed as Equation (8).

(8) $$\begin{array}{c} F_{s}^{\prime }=\frac{1}{\Delta \varphi }=2a_{0}\tau_{r} \end{array}$$

The peak frequency ($F_{sp}^{\prime }$) of the spectrum after resampling is shown in Equation (9),

(9) $$\begin{array}{c} F_{sp}^{\prime }=\frac{F_{s}^{\prime }\cdot k}{N}=\frac{2a_{0}\tau_{r}k}{N} \end{array}$$

where *k* is the peak frequency point. It can be seen from Equation (7) that the nonlinearity of frequency modulation is eliminated.

**Figure 3:**
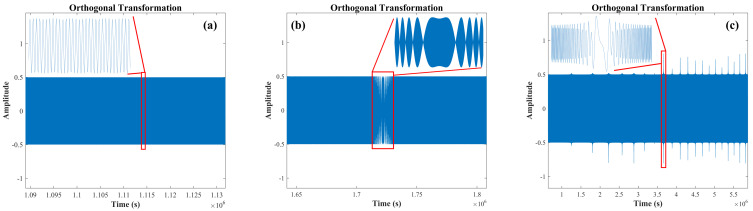
Orthogonal transformation of HT signals: (**a**) OT signal of first order, (**b**) OT signal of second order, (**c**) OT signal of third order.

In order to increase the number of resampled points, HT is used to expand the phase of the beat signal of the auxiliary optical path. However, HT is only applicable to the narrow-band signals. Figure 3 shows the orthogonal transformation of the HT signal. The nonlinear frequency variation coefficient of the laser is within the first order; there is only one oscillatory mode in the orthogonal transformed signal, which is shown in Figure 3a. Figure 3b shows the inconsistent group delay of Hilbert transformed signal at the nonlinear frequency variation of the laser in the second order, which leads to the change of the orthogonal transformed (OT) signal envelop. Furthermore, Figure 3c demonstrates the orthogonal transformed signal which has multiple oscillatory modes at the nonlinear frequency variation of the laser, which extends beyond the third order [27]. HT is not suitable in the case that the nonlinear frequency variation of the laser extends beyond the second order.

**Figure 4:**
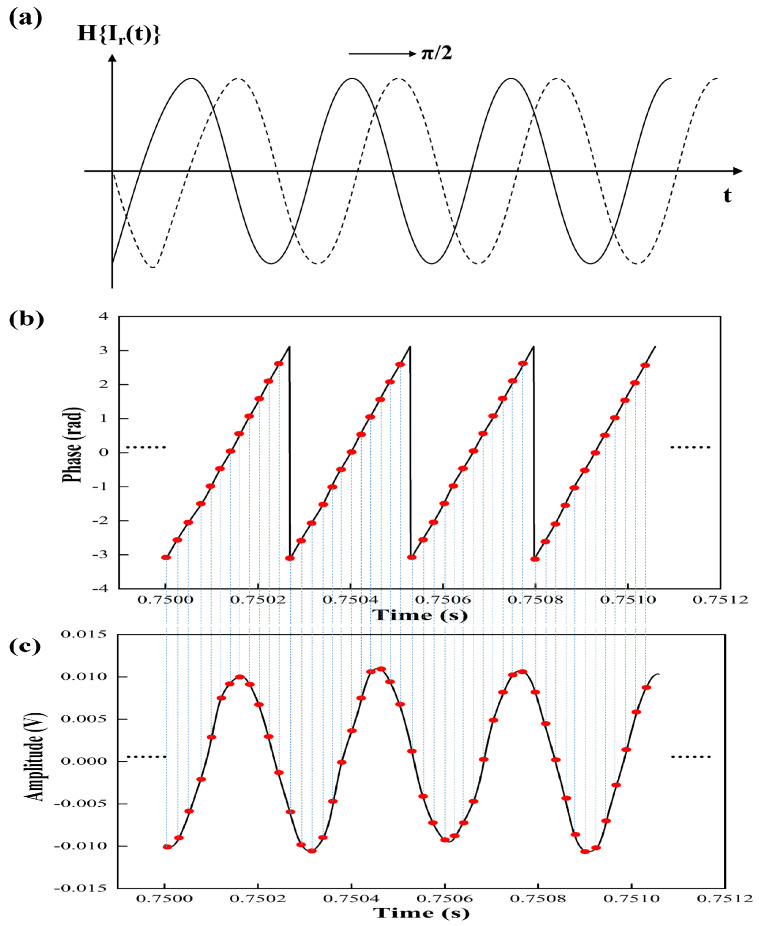
Schematic diagram of HT and resampled signal, (**a**) the beat signal shifting π/2 phase by HT, (**b**) the subdivision phase of beat signal, (**c**) resampled point of beat signal of measurement optical path.

As shown in Figure 4a, the beat signal $I_{r}\left(t\right)$ of auxiliary optical path is transformed π/2 phase by HT, and the dotted line represents the imaginary part *H*$\left\{I_{r}\left(t\right)\right\}$ of the phase [28].

Then, the phase of the beat signal of auxiliary optical path can be expressed as Equation (10).

(10) $$\begin{array}{c} \phi \left(t\right)=\omega \left(t\right)\cdot t+\varphi_{0}=\tan^{-1}\left[H\left\{I_{r}\left(t\right)\right\}/I_{r}\left(t\right)\right] \end{array}$$

The instantaneous phase of the beat signal of the auxiliary optical path with 12 sampled points in each period is shown in Figure 4b. The corresponding resampled point of the beat signal of measurement optical path is shown in the red dot in Figure 4c. The resampled signal can be represented as,

(11) $$\begin{array}{c} I_{sam}^{\prime }=I_{0}\cos\left(2\pi \frac{N\tau_{0}}{2N_{h}\tau_{r}}\right) \end{array}$$

where $N_{h}$ is the resampled point in each period. As long as $2\tau_{0}/N_{h}<\tau_{r}$, the sampling theorem can be satisfied, and resampled signals aliasing will not occur.

The spectrum information of beat signal is usually obtained by FFT. However, FFT is suitable for the periodic stationary signal, which is based on the global information of the signal. To improve the resolution of frequency, it is necessary to increase the global sampled points of the beat signal, which enhances the useless calculation points other than the main frequency. CZT can solve this problem with helix sampling on the unit circle in complex frequency domain, which is suitable to focus on the local characteristics of the signal [29]. The frequency range to be analyzed by CZT is $f_{w}$ ($f_{min}$, $f_{max}$), which is refined into sampling points (M−1). Furthermore, the $f_{min}$ and $f_{max}$ can be expressed as,

(12) $$\begin{array}{c} f_{\min}=\frac{f_{s}\cdot \theta_{0}}{2\pi } \end{array}$$

(13) $$\begin{array}{c} f_{\max}=\frac{f_{s}\cdot \left[\theta_{0}+\left(M-1\right)\varphi_{0}\right]}{2\pi } \end{array}$$

where $f_{s}$ is the sample frequency, *M* represents the number of refined points in the frequency domain (0 < *M* <$L_{res}$), $L_{res}$ is the length of the resample signal, $\theta_{0}$ and $\varphi_{0}$ represent the included angle between adjacent sampling points and the phase of the starting position, respectively. Furthermore, the resolution of frequency by CZT is defined as,

(14) $$\begin{array}{c} R_{czt}=\frac{f_{\max}-f_{\min}}{M-1} \end{array}$$

As long as *M* is large enough, the spectral resolution of CZT can be significantly smaller. We can select appropriate $f_{min}$, $f_{max}$, and *M* to analyze the actual beat signal.

## 3. Experiment and Results

**Figure 5:**
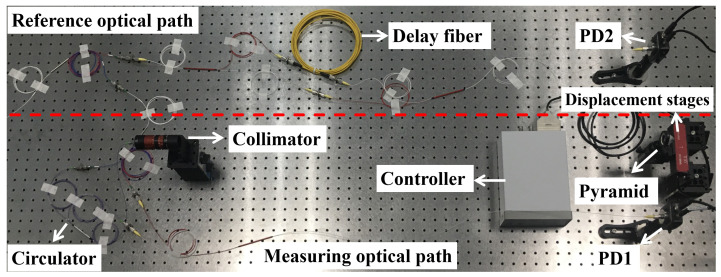
The experimental setup.

To verify the feasibility of this method, we built an FSI ranging system as shown in Figure 5. The light source of the system is the ECDL (New Focus TLB-6700) with a linewidth of 200 kHz. The frequency sweeping range is from 1542.5 nm to 1557.5 nm, and the sweeping speed is 15 nm/s.

The experiments are carried out in a constant temperature and humidity environment. The ranging system is divided into auxiliary optical path and measurement optical path. The change of fiber length caused by temperature change was very small, so the influence of fiber length change on ranging accuracy can be ignored. The beat signals of the measuring optical path and auxiliary optical path are detected by PD1 and PD2 (Thorlabs PDA10CS2). Furthermore, the two beat signals are sampled and recorded by an oscilloscope (Tektronix MSO 70000).

**Figure 6:**
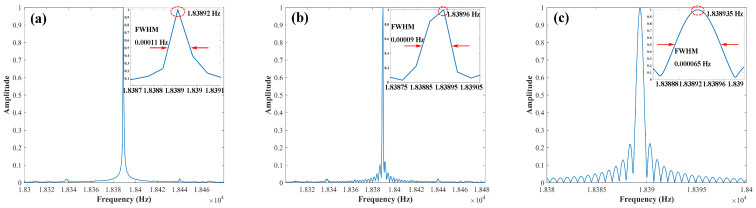
The comparison between FFT and CZT, (**a**) FFT of resampling beat signal without zero padding, (**b**) FFT of resampling beat signal with zero padding, (**c**) CZT of resampling beat signal.

In order to study the time to frequency domain conversion efficiency of FFT and CZT, the same resampling beat signal is processed by FFT and CZT, which is shown in Figure 6. To facilitate the comparison of spectrum information, we normalized the amplitude. The resampling beat single has 222,706 points. Figure 6a displays the result of running the 222,706 points FFT of the resampling beat signal. Figure 6b shows the result of FFT with the padding at zero. The points of FFT are 262,144. Figure 6c demonstrates the result of CZT of resampling the beat signal. Points (*M*) of CZT are 2000. Due to the spectral leakage of FFT, the frequency resolution is limited. However, the CZT is much more flexible with high resolution. Furthermore, the points of CZT are much less than FFT. The resolution can be tailor-made by means of adjusting the start and stop frequencies ($f_{min}$, $f_{max}$) and the number of *M*. Hence, CZT is used to process the resampling beat signal in this research.

**Figure 7:**
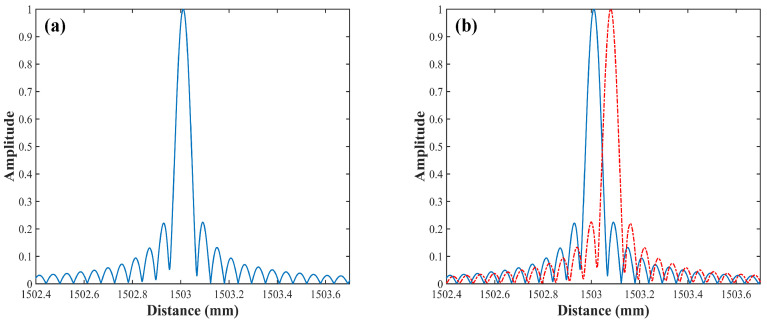
The results of the distance measurements, (**a**) single point ranging resolution, (**b**) single point 70 μm displacement ranging resolution.

Figure 7a shows the distance spectrum with the HT subdivision resampled method and CZT. The optical path difference of the auxiliary path optical is about 3105 mm. Hence, four points are sampled ($N_{h}$ = 4) with an equal frequency interval in a period, and the phase point interval is π/2. The ranging resolution (FWHM) in Figure 7a is 70 μm, which is greatly improved, compared with the result in Figure 2b. In order to provide a quantitative estimation of the resolution enhancement with the proposed technique, the spatial resolution experiment is performed. The pyramid is moved unidirectionally 70 μm on a displacement table (Thorlabs LPS710, which can achieve 800 μm displacement with a step accuracy of 6 nm in the closed-loop working mode). The result of displacement measurement is shown in Figure 7b, where the red curve represents the distance spectrum after displacement, which verifies the ranging resolution of 70 μm. Since the environmental factors (turbulence, vibration, etc.) will affect the results of a single measurement, the average value of multiple measurements can improve the reliability of the ranging results. After measuring 13 times, the standard deviation (σ) is 4.6 μm.

**Figure 8:**
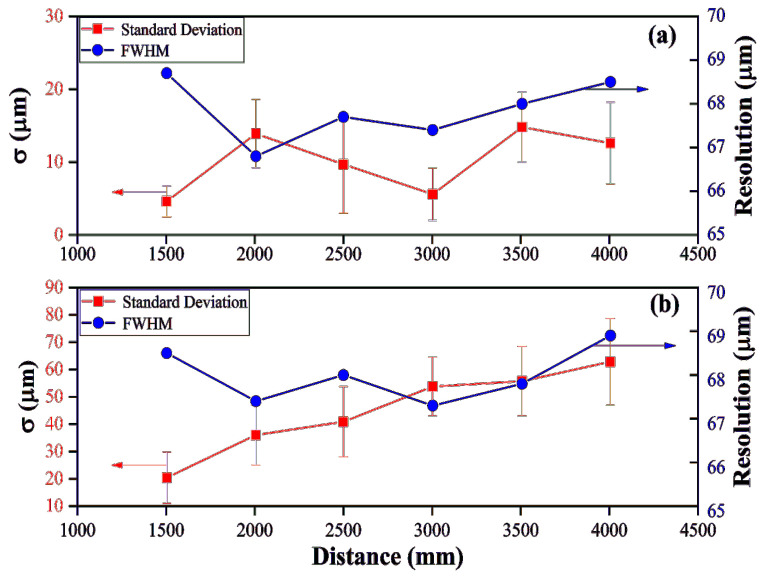
Resolution and standard deviation of different absolute distance measurement, (**a**) different absolute distance measurement results with HT subdivision method, (**b**) different absolute distance measurement results with peak-valley resampling method.

To compare with the ranging accuracy of traditional peak-to-valley resampling methods at different distances, we moved the pyramid by a range of 1.5–4 m. Six different distances are arbitrarily selected and 13 sets of data are taken for each distance. With the HT subdivision resampled method, $N_{h}$ varies with the different measurement distance. The length of the delay fiber in the auxiliary optical path is fixed, and the corresponding $N_{h}$ is set to 4, 6, 7, 8, 10, and 12 respectively to meet the Nyquist sampling theorem. However, the length of the delay fiber in the auxiliary optical path needs to be more than twice as long as that of the measurement optical path with the peak-valley resampling method. Figure 8a,b demonstrates the standard deviation (σ) and resolution of the HT subdivision resampled and the peak-valley resampling, respectively. The red error bar represents the standard error and blue dot is the resolution. The resolution under different measurement distances of two methods is the same. However, σ and the standard error under the HT subdivision resampled method are much less than those of the peak-valley resampling method, which proves that the robustness of the HT subdivision resampled method is higher than the peak-valley resampling method. In other words, the vibration of the environment has a greater impact on variations in the optical path length of long fibers. As for the peak-valley resampling method, the delay fiber in the auxiliary optical path varies with the measurement distance, hence, the σ is gradually increased.

## 4. Discussion

The HT resampling method can refine the equal optical frequency interval in a period. Compared with the peak-valley resampling method used by Pan et al. [30], it significantly reduced the length of the optical fiber and improved the measurement repeatability.

By analyzing the results of HT resampling, the relationship between measurement error and the number of resampled points is found. As shown in Figure 8a, the σ could reach the minimum value (such as the distance of 1500 mm or 3000 mm) at the resampled points meeting the Nyquist sampling theory. When the resampled points are more than the criterion of the Nyquist sampling theory, the σ increases. This agrees well with the observation that more resampled points in each period means larger accumulated sampling errors.

Moreover, the number of points which are sampled by the oscilloscope in each period is limited. The number of resampled points in each period could not keep increasing or the conditions for optical frequency intervals of resampled points could not be met. This leads to the accumulation of sampling errors and affects the repeatability of the distance measurement. Hence, the length of the delay fiber cannot be unlimitedly reduced. The restriction can be lifted by linearly interpolating the auxiliary and measurement beat signals. However, we should further study whether linear interpolation will cause beat signal distortion.

To deal with high-order nonlinear frequency modulation of the ECDL, the Hilbert–Huang transform can be exploited. The empirical mode decomposition of the Hilbert–Huang transform can be used to decompose the interference signal to obtain the intrinsic mode functions and residuals and to further reconstruct the interferometer signal after filtering the high and low frequency. This is worth our future research.

## 5. Conclusions

In this manuscript, we propose an FSI ranging method based on Hilbert transform and Chirp-z transform, which can realize the phase subdivision, the increase in resampled points, and the refinement of frequency points. This method reduces the limit on the length of the delay fiber of the auxiliary optical path in FSI ranging system and shows great advantages in large-scale measurement. The ranging resolution can reach 70 μm and the standard deviation is 12.6 μm over a range of 4005 mm. Compared with traditional peak-valley resampling methods, this has higher robustness. The short delay fiber is beneficial to reducing the impact of vibration on the FSI measurement system, making the FSI system more practical for absolute distance measurement. At the same time, benefiting from the HT algorithm, our method can be easily implemented in all fiber-optic systems, and the system can be configured as a Mach–Zehnder or Michelson interferometer without adding additional devices. Furthermore, this method contributes to the development of FSI laser ranging with the aim of miniaturization and integration.

## Data Availability

All data generated by or that appeared in this study are available upon request to the corresponding author.
